# Comparative Radiologic Outcomes of Romosozumab and Teriparatide in Osteoporotic Vertebral Fractures

**DOI:** 10.3390/jcm15062349

**Published:** 2026-03-19

**Authors:** Jun-Seok Lee, Geon-U Kim, Ho-Young Jung, Young-Hoon Kim, Sang-Il Kim, Sangjun Park, Young-Yul Kim, Hyung-Youl Park

**Affiliations:** 1 Department of Orthopedic Surgery, Eunpyeong St. Mary’s Hospital, College of Medicine, The Catholic University of Korea, Seoul 03312, Republic of Korea; junband@catholic.ac.kr (J.-S.L.); gimgunu4u@naver.com (G.-U.K.); cbjunghy@gmail.com (H.-Y.J.); 2 Department of Orthopedic Surgery, Seoul St. Mary’s Hospital, College of Medicine, The Catholic University of Korea, Seoul 06591, Republic of Korea; boscoa@catholic.ac.kr (Y.-H.K.); sang1kim@catholic.ac.kr (S.-I.K.); jack2020@korea.ac.kr (S.P.); 3 Department of Orthopedic Surgery, Daejeon St. Mary’s Hospital, College of Medicine, The Catholic University of Korea, Daejeon 34943, Republic of Korea; kimtwins72@catholic.ac.kr

**Keywords:** osteoporotic vertebral fracture, romosozumab, teriparatide, bone mineral density, spinal alignment

## Abstract

**Background/Objectives**: Osteoporotic vertebral fractures (OVFs) are frequently associated with progressive kyphotic deformity and vertebral height loss, adversely affecting spinal alignment and function. Although romosozumab and teriparatide are established anabolic therapies, comparative data on their longitudinal radiologic effects after OVFs remain limited. This study compared radiologic and clinical outcomes between these agents in patients with OVFs. **Methods**: Sixty-two patients with single-level OVFs were included: 34 patients in the romosozumab group and 28 patients in the teriparatide group, analyzed in a retrospective, observational comparative study. All patients received anabolic therapy for 6 months followed by sequential denosumab. Radiologic parameters (Cobb angle, vertebral wedge angle, and anterior and middle vertebral body heights) were evaluated at baseline and at 1, 3, 6, and 12 months. Bone mineral density (BMD) and clinical outcomes were assessed at baseline and 12 months. **Results**: Baseline characteristics were comparable between groups. No statistically significant between-group differences were observed in radiologic parameters over 12 months. However, the romosozumab group showed numerically smaller increases in kyphotic angles and less vertebral height loss, particularly beyond 6 months. Lumbar spine BMD increased in both groups, with a greater absolute gain in the romosozumab group. Back pain improved substantially in both groups, while disability improvement was greater in the teriparatide group. **Conclusions**: Romosozumab and teriparatide demonstrated comparable radiologic and clinical outcomes in OVFs. Although differences were not statistically significant, romosozumab showed a numerical trend toward better preservation of spinal alignment and vertebral height.

## 1. Introduction

Osteoporotic vertebral fractures (OVFs) are among the most common fragility fractures in the elderly and represent a major clinical and public health concern [1]. These fractures are frequently associated with acute back pain, progressive spinal deformity, and functional decline, which may result in impaired mobility, reduced quality of life, and increased mortality [2]. In addition to the immediate clinical burden, OVFs may lead to progressive kyphotic deformity and vertebral height loss, particularly at the thoracolumbar junction, thereby influencing long term alignment and mechanical stability [3].

Teriparatide, a recombinant parathyroid hormone (PTH 1-34), has been widely used as an anabolic agent in the treatment of osteoporosis and OVFs [4]. Previous studies have reported that teriparatide may reduce pain, facilitate radiographic fracture healing, and improve functional outcomes in patients with OVFs [5,6,7,8]. However, its clinical use is often limited by the requirement for daily injections and challenges in maintaining long term adherence. In real-world practice, many patients receive teriparatide for a relatively short duration and subsequently transition to antiresorptive therapy [9,10].

Romosozumab is a monoclonal antibody targeting sclerostin and exerts dual effects by stimulating bone formation and suppressing bone resorption [11]. Large, randomized trials have demonstrated that romosozumab rapidly increases bone mineral density (BMD) and reduces the risk of vertebral and non-vertebral fractures [12,13,14]. Despite these favorable effects on bone mass and structure, evidence regarding its role in OVF healing remains limited. In particular, data directly comparing the effects of romosozumab and teriparatide on post-fracture spinal alignment and vertebral height preservation are scarce [15].

Given the clinical importance of maintaining spinal alignment after OVFs, a clearer understanding of how different anabolic agents influence radiologic outcomes is needed. Therefore, the purpose of this study was to compare longitudinal radiologic outcomes between romosozumab and teriparatide in patients with single-level OVFs treated with a standardized sequential regimen. By focusing on changes in spinal alignment and vertebral body height over a 12-month follow-up period, this study aimed to provide clinically relevant insights into the potential impact of anabolic agent selection on post-fracture spinal stability.

## 2. Materials and Methods

### 2.1. Study Design and Patient Selection

This study was designed as a retrospective observational comparative analysis of patients with acute OVFs treated with anabolic agents in a real-world clinical setting. Patients diagnosed with single-level OVFs between January 2022 and December 2023 were considered for inclusion. The study population consisted of a prospectively collected romosozumab cohort and a retrospectively reviewed teriparatide cohort. All included patients were followed for a minimum of 12 months.

Eligible patients met the following criteria: a single-level OVF confirmed by radiography and magnetic resonance imaging, initiation of anabolic therapy within 4 weeks of fracture diagnosis, confirmed by plain radiography combined with CT or MRI, and availability of serial radiographs during follow-up. To minimize confounding from advanced instability or surgical intervention, patients with prior vertebral fractures, pathological fractures, previous spinal surgery, or subsequent operative treatment during follow-up were excluded. Patients who underwent vertebroplasty or kyphoplasty before or during the follow-up period were also excluded.

In the romosozumab group, 56 patients were prospectively screened, and 34 patients met the inclusion criteria. The teriparatide group was derived from a retrospectively reviewed cohort of 187 patients, of whom 28 patients were eligible for final analysis.

This study was approved by the Institutional Review Board of Eunpyeong St. Mary’s Hospital, The Catholic University of Korea (IRB No. PC24RISI0011), and conducted in accordance with the Declaration of Helsinki. As this study was conducted as a retrospective observational analysis, the requirement for informed consent was waived for both groups.

### 2.2. Treatment Protocol and Clinical Management

Romosozumab or teriparatide was initiated during the acute phase following fracture diagnosis. In routine clinical practice at our institution, anabolic therapy is commonly administered for a limited duration because of reimbursement restrictions and patient adherence issues. Therefore, both groups received anabolic therapy for six months, followed by sequential denosumab treatment according to institutional protocol.

Although this treatment duration differs from guideline-recommended courses, this standardized sequence was applied consistently to both groups, allowing a comparative evaluation of early anabolic effects on fracture-related radiologic changes. All patients were managed conservatively without routine external bracing. Analgesics were prescribed as needed according to patient symptoms, but no structured rehabilitation program was mandated [16]. This approach reflects common real-world practice in elderly patients with osteoporotic vertebral fractures.

### 2.3. Baseline Demographic

Baseline demographic and clinical variables included age, sex, body mass index (BMI), diabetes mellitus (DM), hypertension (HTN), dyslipidemia (DLP), hepatitis, inflammatory disease, smoking status, prior osteoporosis treatment, and fracture location (thoracic, thoracolumbar, or lumbar). Previous osteoporosis therapy was categorized as treatment-naïve or as previously treated with selective estrogen receptor modulators (SERMs), bisphosphonates (BPs), denosumab, or parathyroid hormone (PTH).

### 2.4. Radiologic Assessment

Radiologic evaluations were performed at baseline and at 1, 3, 6, and 12 months after fracture diagnosis. Radiologic parameters included Cobb’s angle (CA), vertebral wedge angle (VWA), and anterior (AVH), middle (MVH), and posterior vertebral body heights (PVH):

- CA: angle between the upper endplate of the vertebra above and the lower endplate of the vertebra below the fracture.
- VWA: angle between the upper and lower endplates of the fractured vertebra.
- AVH, MVH, and PVH: perpendicular distances between the corresponding endplates [17].

**Figure 1:**
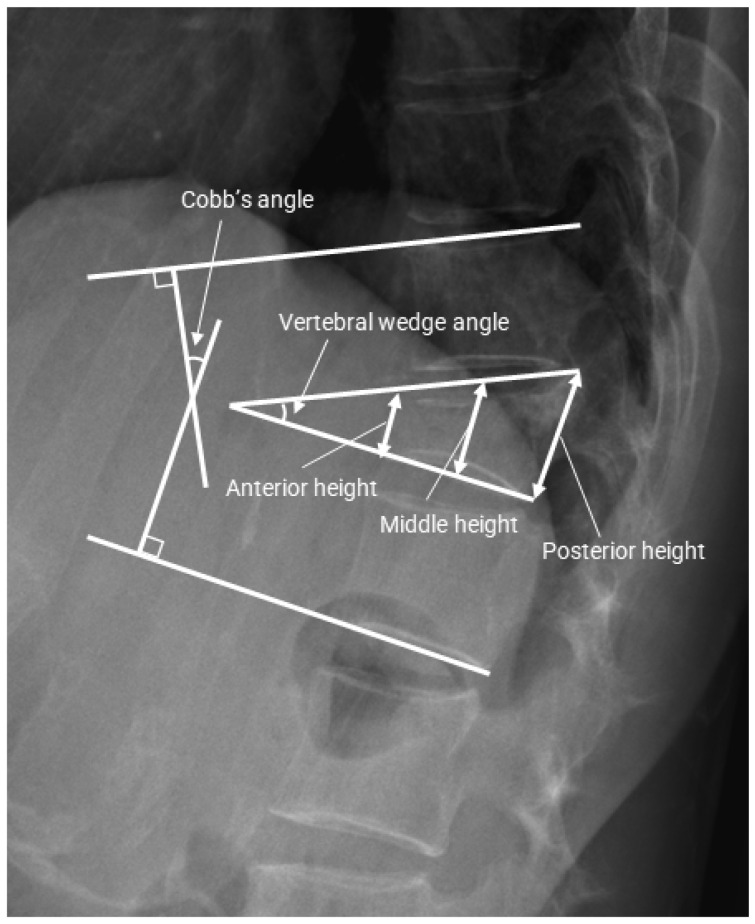
Radiographic parameters used for assessment. Cobb angle (CA), vertebral wedge angle (VWA), anterior vertebral height (AVH), middle vertebral height (MVH), and posterior vertebral height (PVH).

The presence of an intravertebral cleft (IVC) was also assessed on follow-up radiographs [18]. All measurements were independently performed by two spine surgeons, and the mean values were used for analysis. Measurement techniques are illustrated in Figure 1.

### 2.5. BMD and Clinical Parameters

BMD was measured at baseline prior to initiation of anabolic therapy and at the 12-month follow-up using dual-energy X-ray absorptiometry (DXA) (Horizon^®^, Hologic Inc., Marlborough, MA, USA). Measurements included the lumbar spine from L1 to L4, total hip, and femoral neck, with the fractured vertebral excluded to minimize artifact-related inaccuracies [19]. Follow-up measurements were performed at the same anatomical sites to ensure consistency. Both absolute BMD values (g/cm^2^) and T-scores for the spine and femur were used for analysis.

Clinical outcomes were assessed using the numeric rating scale for back pain, right leg pain, and left leg pain, where a score of zero indicated no pain and a score of ten indicated the worst possible pain. Functional disability was evaluated using the Oswestry Disability Index (ODI), expressed as a percentage, with higher scores indicating greater disability. All clinical assessments were performed at baseline and at 12 months. Changes from baseline to follow-up were calculated for all clinical outcome measures.

### 2.6. Study Endpoints

The primary endpoint was the change in radiologic parameters (Cobb angle, vertebral wedge angle, and anterior and middle vertebral body height) at 12 months from baseline. The secondary endpoints included changes in BMD (lumbar spine and femoral neck) and clinical outcomes (NRS back pain score and ODI) at 12 months.

### 2.7. Statistical Analysis

Continuous variables were summarized as means with standard deviations, and categorical variables were presented as counts and percentages. The normality of continuous variables was assessed using the Shapiro–Wilk test. Depending on data distribution, continuous variables were compared between groups using the Student’s *t*-test or the Mann–Whitney U test. Categorical variables were compared using the chi-square test or Fisher’s exact test, as appropriate.

Baseline demographic and clinical characteristics were examined to identify potential imbalances between groups related to the observational study design. Given the presence of baseline differences in lumbar spine BMD, baseline BMD values were included as covariates in all longitudinal analyses, and longitudinal changes in radiologic parameters were analyzed with adjustment for baseline values. Repeated measures analysis of variance was used to evaluate changes in radiologic parameters over time and to explore temporal patterns within and between groups, with baseline radiologic values included as covariates. All analyses were performed using SPSS Statistics (version 26.0; IBM Corp., Armonk, NY, USA). A *p*-value < 0.05 was considered statistically significant.

### 2.8. Use of Artificial Intelligence Tools

The English language of this manuscript was reviewed and edited with the assistance of ChatGPT 5.2 (OpenAI, San Francisco, CA, USA) for grammatical correction purposes only. All scientific content, data analysis, and interpretations were solely the responsibility of the authors.

## 3. Results

### 3.1. Baseline Demographic and Clinical Characteristics

**Figure 2:**
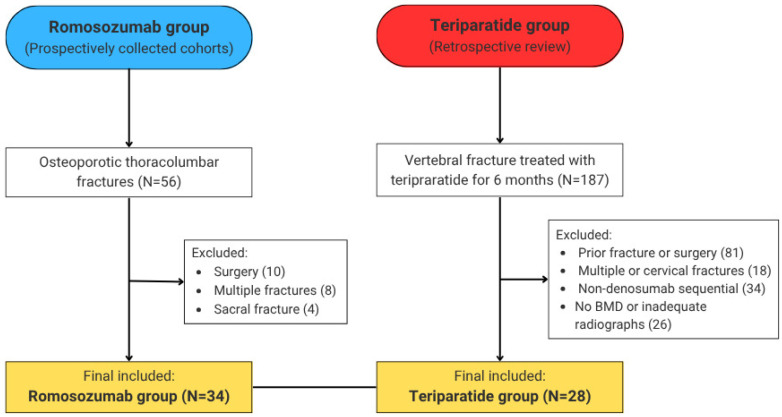
Flow diagram of patient selection. Among 56 patients screened for the romosozumab group and 187 patients screened for the teriparatide group, 34 and 28 patients, respectively, met the inclusion criteria after applying exclusions.

The patient selection process is summarized in Figure 2. Baseline demographic and clinical characteristics are summarized in Table 1. There were no significant differences between the romosozumab and teriparatide groups in terms of age, sex distribution, BMI (24.26 ± 4.31 vs. 24.72 ± 3.85 kg/m^2^, *p* = 0.624), prevalence of DM (26.5% vs. 21.4%, *p* = 0.769), hypertension (50.0% vs. 60.7%, *p* = 0.450), dyslipidemia (14.7% vs. 10.7%, *p* = 0.719), hepatitis (2.9% vs. 3.6%, *p* = 1.000), inflammatory disease (0.0% vs. 3.6%, *p* = 0.452), or smoking status. The etiology of osteoporosis was predominantly age-related (primary osteoporosis) in both groups, with no patients receiving glucocorticoids or medications known to cause secondary osteoporosis. All patients received calcium and vitamin D supplementation as standard care.

The proportion of treatment-naïve patients was comparable between the groups (67.6% vs. 82.1%, *p* = 0.324). The distribution of previously used agents, including SERM, BP, denosumab, and PTH, did not differ significantly.

Fracture site distribution was also similar between the groups, with no significant differences in the proportions of thoracic, thoracolumbar, and lumbar fractures (*p* = 0.506).

### 3.2. Radiologic Outcomes Between the Two Groups

**Figure 3:**
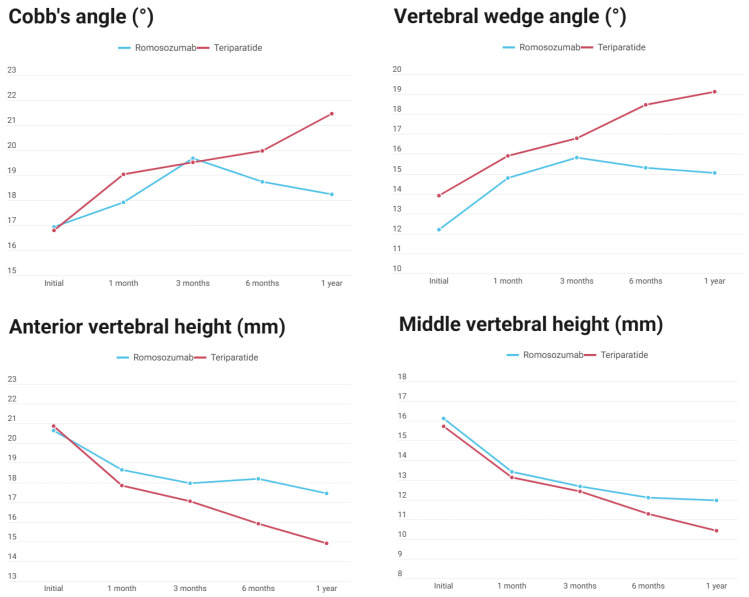
Serial changes in radiologic outcomes over 12 months in the romosozumab and teriparatide groups. Cobb angle (CA), vertebral wedge angle (VWA), anterior vertebral height (AVH), and middle vertebral height (MVH).

Serial radiologic outcomes are summarized in Table 2 and illustrated in Figure 3. At baseline, there were no statistically significant differences between the romosozumab and teriparatide groups in CA, VWA, and vertebral body height parameters, including AVH, MVH, and PVH.

During follow-up at 1, 3, 6, and 12 months, radiologic parameters did not differ significantly between the two groups at any time point. However, longitudinal analysis demonstrated a numerical trend favoring the romosozumab group with respect to maintenance of spinal alignment and preservation of vertebral body height. At 12 months, the increase from baseline in Cobb angle was smaller in the romosozumab group compared with the teriparatide group (Δ2.08° vs. Δ5.22°). A similar pattern was observed for vertebral wedge angle, with a smaller increase in the romosozumab group (Δ3.19° vs. Δ4.79°).

Loss of AVH was less pronounced in the romosozumab group compared with the teriparatide group (−3.36 mm versus −5.17 mm). MVH also showed a similar tendency, although the difference between groups was small (−4.62 mm versus −4.75 mm). At the 12-month follow-up, VWA was numerically lower in the romosozumab group compared with the teriparatide group (15.05° versus 19.13°), showing a tendency toward statistical significance (*p* = 0.054).

Repeated measures ANOVA revealed no significant time-by-group interaction for CA (*p* = 0.274), VWA (*p* = 0.143), AVH (*p* = 0.276), or MVH (*p* = 0.411). Nevertheless, graphical trends in Figure 3 showed a numerical trend toward better maintenance of vertebral alignment and vertebral height with romosozumab, particularly at 6 and 12 months. The incidence of IVC was comparable between the groups (11.8% vs. 14.3%, *p* = 1.000).

### 3.3. BMD Outcomes

Changes in bone mineral density over the 12-month follow-up period are summarized in Table 3. At baseline, lumbar spine bone mineral density was significantly lower in the romosozumab group compared with the teriparatide group. Femoral bone mineral density did not differ significantly between groups at baseline.

At 12 months, lumbar spine bone mineral density increased in both groups. The absolute increase was greater in the romosozumab group (0.065 ± 0.068 g/cm^2^) compared with the teriparatide group (0.051 ± 0.036 g/cm^2^), although this difference did not reach statistical significance. The percentage increase from baseline showed a similar pattern. In contrast, changes in femoral bone mineral density were minimal in both groups, with no statistically significant difference between groups.

### 3.4. Safety Outcomes

No serious adverse events directly attributable to either romosozumab or teriparatide were recorded during the 6-month anabolic therapy period or subsequent denosumab treatment. Specifically, no cases of cardiovascular events, hypocalcemia, or injection-site reactions requiring treatment discontinuation were observed in either group.

### 3.5. Clinical Outcomes Between the Two Groups

**Figure 4:**
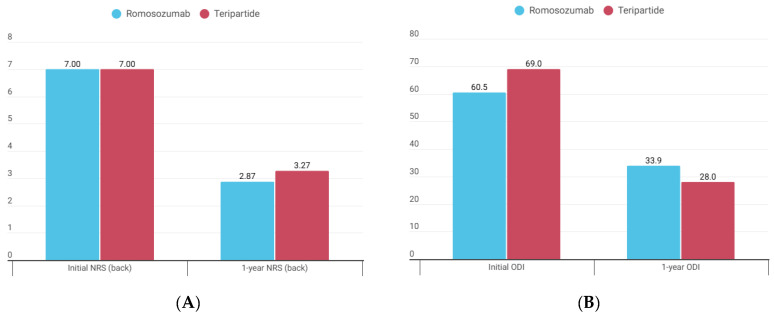
Clinical outcomes over 12 months in the romosozumab and teriparatide groups. (**A**) Numeric rating scale (NRS) for back pain; (**B**) Oswestry Disability Index (ODI).

Clinical outcomes are summarized in Table 4. For NRS back pain, scores improved from 7.00 ± 1.32 at baseline to 2.87 ± 1.64 at 12 months in the romosozumab group, and from 7.00 ± 1.83 to 3.27 ± 1.39 in the teriparatide group. The degree of reduction was similar between groups (−4.07 ± 1.83 vs. −3.87 ± 1.92, *p* = 0.967) (Figure 4A). NRS scores for right and left leg pain were low at baseline and showed no significant changes in either group during follow-up.

Regarding functional outcomes, the ODI improved in both groups over the 12-month follow-up period. The mean reduction in ODI score was significantly greater in the teriparatide group compared with the romosozumab group (−40.5 ± 16.3 percent versus −26.7 ± 14.3 percent, *p* = 0.020) (Figure 4B).

## 4. Discussion

Previous studies have consistently demonstrated that teriparatide enhances fracture healing in patients with OVFs, particularly when compared with antiresorptive agents [7,20]. Through its anabolic action, teriparatide has been shown to accelerate fracture union and improve early clinical outcomes [15,21]. In our previous study on acute osteoporotic spinal fractures, teriparatide treatment was associated with reduced vertebral height loss (Odds ratio = 0.325) and greater pain improvement compared with both the bisphosphonate-treated patients and untreated controls (NRS change: 5.7 vs. 3.1 and 3.5; *p* < 0.001), supporting its role in enhancing fracture healing and early clinical recovery [6]. Despite this established evidence, data comparing teriparatide with newer anabolic agents in the context of post fracture spinal alignment remain limited, providing the rationale for the present comparative analysis.

In contrast to teriparatide, evidence regarding the effects of romosozumab on fracture healing is relatively sparse. Recent randomized controlled trials evaluating romosozumab in hip and tibial diaphyseal fractures reported no significant improvements in clinical or radiographic outcomes compared with placebo [22,23]. However, these studies focused primarily on cortical bone fractures. Vertebral bodies consist predominantly of trabecular bone, which is metabolically more active and generally more responsive to anabolic stimulation [24,25]. Unlike diaphyseal cortical fractures, vertebral fractures occur in cancellous bone, where healing is primarily mediated by intramembranous and inter-trabecular bone formation. The rich vascularity and favorable biological environment of the vertebral body may therefore allow greater responsiveness to anabolic agents, suggesting that the effects of romosozumab on vertebral fractures may differ from those observed in cortical bone injuries [26,27].

In the present study, radiologic outcomes demonstrated a numerical trend toward better preservation of vertebral body height and spinal alignment in patients treated with romosozumab. Although no statistically significant between-group differences were identified in longitudinal analyses, changes in alignment parameters and vertebral body morphology showed a time-dependent pattern that favored romosozumab, particularly after the acute phase of fracture healing. These findings suggest that romosozumab may contribute to the maintenance of post-fracture spinal alignment.

However, it should be noted that anabolic therapy addresses the biological aspect of fracture healing, and mechanical stability remains a critical consideration in OVF management. In cases where pharmacological treatment alone may be insufficient due to high mechanical instability, surgical stabilization represents an important complementary option. The use of cannulated fenestrated screws augmented with polymethylmethacrylate (PMMA) cement has been reported to show significant long-term effectiveness in maintaining radiological correction and reducing disability in patients with osteoporotic vertebral fractures [28].

The thoracolumbar junction, located at the transition between lumbar lordosis and thoracic kyphosis, is an area prone to progressive kyphosis and complications [29]. In the subgroup analysis of thoracolumbar junction fractures (Supplement Table S1), patients treated with romosozumab exhibited greater baseline deformity. Despite this disadvantage, final alignment parameters at 12 months were comparable between groups, with a tendency toward less progression of kyphotic deformity in the romosozumab group. This observation further supports the potential role of romosozumab in mitigating progressive alignment deterioration in high-risk fracture locations.

These favorable radiologic trends were observed despite the romosozumab group having a lower baseline spine T-score (−2.83 vs. −2.01). Although not statistically significant, the observed numerical trends suggest that romosozumab may contribute to better maintenance of spinal alignment and vertebral height following OVFs.

Similarly, in a previous study directly comparing romosozumab and teriparatide in patients with OVFs, Park et al. [15] reported that the romosozumab group demonstrated lower changes in compression ratio (4.3% vs. 6.4%) and CA (1.0° vs. 2.7°) at 12 months compared to the teriparatide group, showing a similar trend to our findings.

From a mechanistic perspective, quantitative CT studies have demonstrated that romosozumab induces rapid and widespread bone formation within the vertebral body, involving both trabecular and cortical compartments, with significantly greater gains compared with teriparatide [30,31]. This accelerated fracture consolidation may help prevent delayed kyphotic changes, as reflected in the smaller progression of kyphosis observed after 6 months in the romosozumab group [32]. In patients with thoracolumbar fractures, despite lower baseline BMD and greater initial kyphosis, early stabilization of the fracture with romosozumab appeared to preserve spinal alignment without further progression of kyphosis. These observations are consistent with previous reports showing that romosozumab treatment increases Hounsfield unit values at instrumented levels, which were associated with a reduced incidence of proximal junctional kyphosis, supporting its role in enhancing vertebral strength and stability [33].

With regard to BMD, spine values and T-scores were lower at baseline in the romosozumab group; nevertheless, the increase in spine BMD at 12 months was greater with the romosozumab group than with teriparatide (0.065 vs. 0.051). Prior QCT-based studies reported that romosozumab increases both trabecular and cortical volumetric BMD at the spine and hip, with particularly greater cortical gains compared with teriparatide [30]. Accordingly, an increase in hip BMD is generally expected with romosozumab therapy [34]. In this study, however, femoral BMD improved more in the teriparatide group (0.010 vs. −0.005), although this difference was not significant. The absence of femoral BMD improvement in the romosozumab group may be related to the small sample size and the higher prevalence of prior osteoporosis treatment before initiation of therapy [35].

Although both groups received anabolic therapy for six months, which is shorter than the guideline-recommended durations of 12 months for romosozumab and 24 months for teriparatide, this reflects real-world reimbursement constraints in Korea [36]. Since both groups received identical treatment durations, the comparative findings remain valid within this framework, though the observed trends may represent an underestimate of the true between-group differences.

In terms of clinical outcomes, there were no significant differences between the two groups in NRS for back pain, NRS for leg pain, or ODI at each time point. Pain reduction, as measured by NRS for back pain, was also similar (−4.07 vs. −3.87). In a previous study directly comparing teriparatide and romosozumab, serial NRS assessments showed no significant differences at individual time points, but the overall reduction was significantly greater in the romosozumab group (−6.6 ± 2.0 vs. −5.5 ± 2.1, *p* = 0.013), suggesting a more notable improvement in pain relief [15].

A recent network meta-analysis further demonstrated that daily or weekly teriparatide administration was associated with superior long-term pain reduction compared with BPs, with efficacy comparable to non-steroidal anti-inflammatory drugs (NSAIDs) for nonspecific pain control [7]. In this context, our finding that romosozumab achieved pain relief comparable to that of teriparatide is encouraging. This suggests the possibility that romosozumab, in addition to its rapid effects on bone mass and structure, may also provide meaningful analgesic benefits in OVFs. Supporting this view, Mun et al. [37] reported that romosozumab treatment resulted in greater back pain reduction and lumbar spine BMD improvement at 12 months compared with vertebroplasty, suggesting both its analgesic and anabolic potential in OVFs.

With respect to functional outcomes, the reduction in ODI was greater in the teriparatide group (−26.7% vs. −40.5%). However, this finding should be interpreted with caution. First, the baseline ODI was higher in the teriparatide group (69.0 vs. 60.5), which may have contributed to greater absolute improvement through a floor effect. Second, ODI primarily reflects disability related to lumbar spine disorders, and the observed difference may have been affected by concomitant conditions such as spinal stenosis or other spinal pathologies that could not be fully excluded [38]. Importantly, the magnitude of ODI improvement in both groups exceeded the minimal clinically important difference (MCID, 12.8 points), indicating that clinically meaningful functional recovery was achieved regardless of the treatment agent [39].

This study has several limitations. First, the sample size of *N* = 62 is relatively small, which limits statistical power to detect modest between-group differences and may explain the absence of statistically significant findings despite observed numerical trends. Future prospective randomized studies with larger sample sizes are warranted to confirm these findings. Second, the non-randomized, observational nature of this study introduces potential selection bias. Although strict inclusion and exclusion criteria were applied consistently to both groups, the possibility of residual confounding cannot be fully excluded. Third, both anabolic agents were administered for only six months, which is shorter than the recommended durations of 12 months for romosozumab and 24 months for teriparatide. This reflects real-world reimbursement constraints in Korea and may have attenuated the full anabolic potential of both agents [36]. The long-term radiologic outcomes observed at 12 months may therefore not fully reflect the effects achievable with complete guideline-recommended courses. Fourth, the absence of complete biochemical bone turnover marker data (P1NP and CTX) across all patients limited our ability to provide mechanistic support for the observed radiologic changes. Fifth, the follow-up period was limited to 12 months, precluding the evaluation of subsequent fractures or longer-term changes in BMD. Sixth, clinical outcomes in the teriparatide group were assessed retrospectively, raising the potential for recall bias. Seventh, this study lacked a control group. However, its primary focus was to compare the effects of two anabolic agents, and this design reflects the existing literature [40]. Finally, baseline differences existed between the groups: the romosozumab cohort had lower spine BMD and, although not statistically significant, a higher proportion of prior use of osteoporosis medication.

These limitations should be addressed in future prospective studies with longer treatment durations, extended follow-up, and larger, well-controlled cohorts to confirm our findings. In particular, given that radiological outcomes did not reach statistical significance, and hip BMD and functional outcomes differed from previous studies, further investigation is warranted to clarify these discrepancies.

Nevertheless, this study has several notable strengths. A standardized treatment protocol was applied, in which patients with vertebral compression fractures received either romosozumab or teriparatide for six months, followed by a transition to denosumab. To our knowledge, this is the first study to longitudinally assess multiple radiologic parameters to evaluate spine fracture healing under anabolic therapy. By demonstrating the potential benefits of romosozumab in vertebral fracture management, this study provides valuable insights that may guide both future research and clinical practice.

## 5. Conclusions

Both romosozumab and teriparatide demonstrated comparable radiologic and clinical outcomes in the management of OVFs. Although between-group differences did not reach statistical significance, romosozumab showed a numerical trend toward better maintenance of spinal alignment and vertebral body morphology, particularly beyond the acute phase of fracture healing. In thoracolumbar junction fractures, alignment was preserved with romosozumab despite greater initial deformity. These findings suggest that romosozumab may offer potential radiologic advantages in vertebral fractures, warranting further investigation in larger prospective studies with longer follow-up.

## Figures and Tables

**Table 1:** Baseline characteristics of the patients.

	Romosozumab (*n* = 34)	Teriparatide (*n* = 28)	*p*-Value
**Age**	74.7 ± 8.0	73.8 ± 11.6	0.724
**Sex (M:F)**	7 (20.6%): 27 (79.4%)	6 (21.4%): 22 (78.6%)	1.000
**BMI (kg/m^2^)**	24.26 ± 4.31	24.72 ± 3.85	0.624
**DM (%)**	9 (26.5%)	6 (21.4%)	0.870
**HTN (%)**	17 (50.0%)	17 (60.7%)	0.557
**Dyslipidemia (%)**	5 (14.7%)	3 (10.7%)	0.719
**Hepatitis (%)**	1 (2.9%)	1 (3.6%)	1.000
**Inflammatory disease (%)**	0 (0.0%)	1 (3.6%) Ankylosing spondylitis	0.452
**Smoking (%)**	0 (0%)	0 (0%)	1.000
**Previous osteoporosis**			
Naive	23 (67.6%)	23 (82.1%)	0.324
SERM	1 (2.9%)	0 (0%)	
BP	7 (20.6%)	2 (7.1%)	
Denosumab	1 (2.9%)	3 (10.7%)	
PTH	2 (5.9%)	(0%)	
**Fracture site**			
Thoracic (~D10)	2 (5.9%)	1 (3.6%)	0.506
Thoracolumbar (D11-L1)	16 (47.1%)	18 (64.3%)	
Lumbar (L2~)	16 (47.1%)	9 (32.1%)	

M = male, F = female, BMI = body mass index, DM = diabetes mellitus, HTN = hypertension, SERM = selective estrogen receptor modulator, BP = bisphosphonate, PTH = parathyroid hormone, D = thoracic, L = lumbar.

**Table 2:** Serial radiologic outcomes between the romosozumab and teriparatide groups.

	Romosozumab (*n* = 34)	Teriparatide (*n* = 28)	*p*-Value
**Cobb’s angle (°)**			
Initial	16.94 ± 12.90	16.79 ± 8.96	0.843 *
1 month	17.91 ± 11.57	19.04 ± 8.27	0.676
3 months	19.68 ± 12.20	19.52 ± 10.11	0.955
6 months	18.74 ± 12.43	19.96 ± 10.06	0.686
1 year	18.24 ± 11.38	21.46 ± 11.03	0.315
Change from baseline	2.08 ± 5.81	5.22 ± 6.74	0.180
**Vertebral wedge angle (°)**			
Initial	12.19 ± 7.73	13.89 ± 5.97	0.343
1 month	14.79 ± 7.96	15.89 ± 7.40	0.587
3 months	15.82 ± 7.38	16.78 ± 8.25	0.635
6 months	15.29 ± 8.13	18.46 ± 7.38	0.127
1 year	15.05 ± 7.90	19.13 ± 6.57	0.054
Change from baseline	3.19 ± 4.13	4.79 ± 5.33	0.237
**Anterior vertebral body height (mm)**			
Initial	20.64 ± 4.94	20.86 ± 5.42	0.867
1 month	18.65 ± 5.88	17.85 ± 5.83	0.602
3 months	17.95 ± 5.89	17.04 ± 6.35	0.565
6 months	18.19 ± 6.02	15.92 ± 5.99	0.157
1 year	17.44 ± 6.52	14.92 ± 6.11	0.166
Change from baseline	−3.36 ± 3.39	−5.17 ± 3.94	0.088
**Middle vertebral body height (mm)**			
Initial	16.11 ± 3.78	15.71 ± 5.00	0.725
1 month	13.41 ± 4.26	13.12 ± 5.29	0.811
3 months	12.68 ± 4.28	12.41 ± 5.06	0.823
6 months	12.10 ± 4.50	11.27 ± 5.46	0.521
1 year	11.95 ± 5.09	10.42 ± 5.20	0.298
Change from baseline	−4.62 ± 4.29	−4.75 ± 4.48	0.914
**Posterior vertebral body height (mm)**			
Initial	28.12 ± 4.35	28.71 ± 3.32	0.727 *
1 month	27.88 ± 4.11	27.39 ± 3.15	0.485 *
3 months	27.83 ± 4.30	27.30 ± 3.06	0.590
6 months	27.47 ± 4.37	27.35 ± 3.19	0.904
1 year	27.22 ± 4.61	27.42 ± 3.75	0.872
Change from baseline	−0.81 ± 3.24	−1.50 ± 2.92	0.432
**IVC**			
No IVC	30 (88.2%)	24 (85.7%)	1.000
IVC	4 (11.8%)	4 (14.3%)	

IVC = intervertebral cleft. * *p*-values were analyzed using the Mann–Whitney U test.

**Table 3:** Changes in BMD and T-scores between the romosozumab and teriparatide groups.

	Romosozumab (*n* = 34)	Teriparatide (*n* = 28)	*p*-Value
**Lumbar spine area (cm^2^)**			
Initial	43.43 ± 11.02	46.64 ± 9.53	0.349
1 year	43.28 ± 10.67	46.87 ± 9.47	0.338
**Lumbar spine BMD (g/cm^2^)**			
Initial	0.694 ± 0.126	0.773 ± 0.121	0.034
1 year	0.759 ± 0.100	0.824 ± 0.140	0.066
Change from baseline	0.065 ± 0.068	0.051 ± 0.036	0.110 *
Change from baseline (%)	10.74% ± 10.48	6.47% ± 4.09	0.058
**Lumar spine T-score**			
Initial	−2.83 ± 1.01	−2.01 ± 1.23	0.010 *
1 year	−2.08 ± 0.93	−1.53 ± 1.23	0.085
**Femur BMD (g/cm^2^)**			
Initial	0.569 ± 0.116	0.604 ± 0.097	0.256
1 year	0.563 ± 0.088	0.614 ± 0.084	0.046
Change from baseline	−0.005 ± 0.071	0.010 ± 0.035	0.516 *
Change from baseline (%)	1.38% ± 16.23	2.12% ± 6.30	0.461 *
**Femur T-score**			
Initial	−2.42 ± 0.92	−1.90 ± 0.92	0.055 *
1 year	−2.34 ± 0.73	−1.81 ± 0.75	0.016

BMD = bone mineral density. * *p*-values were analyzed using the Mann–Whitney U test.

**Table 4:** Changes in clinical outcomes between the romosozumab and teriparatide groups.

	Romosozumab (*n* = 34)	Teriparatide (*n* = 28)	*p*-Value
**NRS (back)**			
Initial	7.00 ± 1.32	7.00 ± 1.83	1.000
1 year	2.87 ± 1.64	3.27 ± 1.39	0.477
Change from baseline	−4.07 ± 1.83	−3.87 ± 1.92	0.967 *
**NRS (right leg)**			
Initial	0.63 ± 1.54	0.31 ± 1.01	0.752 *
1 year	0.60 ± 0.99	0.60 ± 1.24	0.713 *
Change from baseline	−0.07 ± 1.53	0.27 ± 0.96	0.775 *
**NRS (left leg)**			
Initial	1.13 ± 2.00	0.31 ± 1.01	0.323 *
1 year	0.53 ± 0.92	0.20 ± 0.78	0.250 *
Change from baseline	−0.67 ± 1.96	−0.13 ± 1.19	0.412 *
**ODI (%)**			
Initial	60.5 ± 13.9	69.0 ± 10.7	0.062
1 year	33.9 ± 15.7	28.0 ± 12.4	0.266
Change from baseline	−26.7 ± 14.3	−40.5 ± 16.3	0.020

NRS = numeric rating scale, ODI = Oswestry disability index. * *p*-values were analyzed using the Mann–Whitney U test.

## Data Availability

The datasets used and/or analyzed during the current study are available from the corresponding author upon reasonable request.

## Supplementary Materials

The following supporting information can be downloaded at: https://www.mdpi.com/article/10.3390/jcm15062349/s1. Table S1: Serial radiologic outcomes in patients with osteoporotic vertebral fractures at the thoracolumbar junction.

## Abbreviations

The following abbreviations are used in this manuscript:

OVF	Osteoporotic vertebral fracture
BMD	Bone mineral density
DM	Diabetes mellitus
SERM	Selective estrogen receptor modulator
BP	Bisphosphonate
PTH	Parathyroid hormone
CA	Cobb’s angle
VWA	Vertebral wedge angle
AVH	Anterior vertebral body height
MVH	Middle vertebral body height
PVH	Posterior vertebral body height
IVC	Intravertebral cleft
DXA	Dual-energy X-ray absorptiometry
NRS	Numeric rating scale
ODI	Oswestry Disability Index
ANOVA	Analysis of variance
NSAIDs	Non-steroidal anti-inflammatory drug
MCID	Minimal clinically important difference
