# Supplementary material for: Comparative Radiologic Outcomes of Romosozumab and Teriparatide in Osteoporotic Vertebral Fractures
Source: J Clin Med. 2026 Mar 19;15(6):2349. doi: 10.3390/jcm15062349 (PMC13027127; doi:10.3390/jcm15062349)
Supplement: Supplementary file 1 [file jcm-15-02349-s001.zip › jcm-4170769-supplementary.pdf]

Supplement Table S1. Serial radiologic outcomes in patients with osteoporotic vertebral fractures at the thoracolumbar junction

	**Romosozumab (n=16)**	**Teriparatide (n=18)**	***p*-value**
**Cobb's angle (°)**			
Initial	19.69 ± 7.69	15.50 ± 9.34	0.166
1 month	23.00 ± 8.00	20.63 ± 8.21	0.414
3 months	24.52 ± 8.28	21.94 ± 10.46	0.441
6 months	24.90 ± 9.28	20.94 ± 9.70	0.243
1 year	23.83 ± 10.49	22.17 ± 10.61	0.675
Change from baseline	5.66 ± 4.75	6.63 ± 6.71	0.669
**Vertebral wedge angle (°)**			
Initial	16.96 ± 5.02	15.50 ± 4.81	0.392
1 month	20.01 ± 5.28	18.44 ± 5.25	0.406
3 months	20.94 ± 5.04	19.59 ± 6.15	0.498
6 months	21.49 ± 3.86	20.06 ± 6.09	0.438
1 year	20.39 ± 5.44	19.94 ± 5.74	0.833
Change from baseline	3.67 ± 4.78	4.44 ± 5.76	0.702
**Anterior vertebral body height (mm)**			
Initial	18.23 ± 4.44	19.72 ± 3.61	0.287
1 month	15.50 ± 3.81	15.88 ± 3.59	0.777
3 months	14.83 ± 4.09	15.18 ± 4.67	0.820
6 months	14.54 ± 3.94	14.33 ± 4.13	0.885
1 year	13.53 ± 3.87	13.94 ± 4.72	0.851*
Change from baseline	-5.19 ± 2.98	-5.78 ± 4.07	0.672
**Middle vertebral body height (mm)**			
Initial	15.92 ± 3.95	15.61 ± 3.99	0.823
1 month	12.31 ± 3.14	12.25 ± 4.01	0.961
3 months	11.44 ± 3.67	11.41 ± 3.91	0.984
6 months	11.10 ± 3.68	10.28 ± 4.64	0.583
1 year	9.98 ± 3.81	9.61 ± 4.15	0.810
Change from baseline	-6.67 ± 3.72	-6.00 ± 4.37	0.668
**Posterior vertebral body height (mm)**			
Initial	29.1 ± 4.32	29.17 ± 2.48	0.972
1 month	28.0 ± 4.24	27.25 ± 2.08	0.530
3 months	28.1 ± 4.16	27.18 ± 2.19	0.411
6 months	27.97 ± 4.35	27.06 ± 2.13	0.439
1 year	27.07 ± 5.10	26.89 ± 2.87	0.904
Change from baseline	-2.25 ± 3.67	-2.28 ± 2.78	0.981
**IVC**			
No IVC	13 (81.2%)	14 (77.8%)	0.571
IVC	3 (18.8%)	4 (22.2%)	

IVC = intervertebral cleft. **p*-values were analyzed using the Mann–Whitney U test.
